# Calcifications pelviennes: du bénin au malin

**DOI:** 10.11604/pamj.2015.22.86.7821

**Published:** 2015-10-01

**Authors:** Haykel Abdelhedi, Naziha Khammassi

**Affiliations:** 1 Faculté de Médecine de Tunis, Service de Médecine Interne, Hôpital Razi, 2010, La Manouba, Tunisie

**Keywords:** Fibrome utérin, fibromes calcifiés, calcifications pelviennes, Uterine fibroids, calcified fibroids, pelvic calcifications

## Image en medicine

Les fibromes ou myomes utérins sont des tumeurs bénignes encapsulées, constituées de tissu musculaire utérin. Ce sont des lésions fréquentes retrouvées dans près de 20% des cas chez des femmes de plus de 35 ans. Plus rarement, une femme jeune peut développer un fibrome. Nous rapportons l'observation d'une patiente âgée de 54 ans, ménopausée depuis 6 ans, hospitalisée pour exploration d'une mononeuropathie multiple sensitivo-motrice axonale associée à une neuropathie végétative à type de diarrhée motrice. Au cours de son hospitalisation la patiente a présenté une hématurie macroscopique totale. L'examen cytobactériologique des urines a éliminé une infection urinaire. Un arbre urinaire sans préparation a objectivé une opacité pelvienne de tonalité calcique hétérogène faisant 60x30mm. L’échographie abdomino-pelvienne a permis d’écarter une lithiase ou une tumeur des voies urinaires. L'examen gynécologique spécialisé a conclu à un fibrome utérin calcifié. La conduite à tenir était l'abstention thérapeutique. Devant une calcification pelvienne les principales étiologies sont essentiellement représentées par les tératomes ovariens, les fibromes calcifiés, les tumeurs urinaires ou digestives, les calcifications épiploïques et exceptionnellement le lithopédion qui est une forme particulière et rare de grossesse extra-utérine d’évolution chronique.

**Figure 1:**
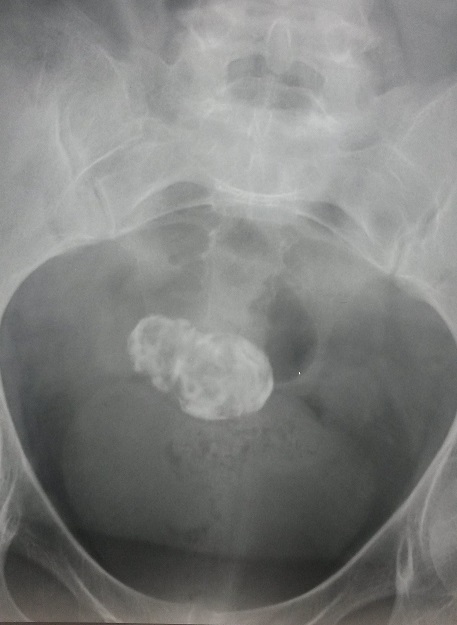
Arbre urinaire sans préparation: opacité pelvienne de tonalité calcique hétérogène faisant 60x30mm

